# Variety in mental health research data: when does more become too much?

**DOI:** 10.1186/1471-244X-7-45

**Published:** 2007-09-05

**Authors:** Jan Luijsterburg, Joop van den Bogaard, Pieter de Vries Robbé

**Affiliations:** 1 Tranzo, Scientific Center for Transformation in Care and Welfare, Tilburg University, PO box 90153, 5000 LE Tilburg, the Netherlands; 2 Stichting GGZ Midden-Brabant, PO box 770, 5000 AT Tilburg, The Netherlands; 3 Department of Medical Informatics, Radboud University Nijmegen Medical Centre, PO box 9101, 6500 HB Nijmegen, the Netherlands

## Abstract

**Background:**

Institutes for mental health care consider scientific research an important activity. A good way to stimulate research is by simplifying data collection. Creating a minimal data set for research purposes would be one way to achieve this, however, this would only be possible if the researchers use a limited variety of data types. This article will address the question whether or not this is the case.

**Methods:**

Researchers working in Dutch mental health institutes were approached and asked to complete an internet questionnaire on the individual variables they collected for, and measurement instruments used in, their studies.

**Results:**

In the 92 studies described by the researchers, 124 different variables were collected, and 223 different instruments were used. A total of 66% of the variables and 73% of the instruments were only used in one study.

**Conclusion:**

There is little commonality among research data, hence flexibility will be a crucial factor in facilitating data collection for research in mental health institutes. Nevertheless, reducing the variety of variables and instruments used is important to increase the comparability of results.

## Background

Mental health institutes are becoming increasingly aware of the necessity to support their activities using practice-based scientific research. The reasoning behind this is that results of this research will benefit the quality of treatments, as well as subsequent knowledge about psychiatric illnesses and the effectiveness and efficiency of interventions or programs. Moreover, health care workers who carry out research could develop stronger observation skills for detecting (side) effects, greater insight into patient well-being, and a more organized way of treating patients. Practice-based research is strongly supported by organizations of mental health consumers, governments, and insurance companies, who increasingly hold institutes accountable for both the cost-efficiency and the quality of care provided [1].

Sackett et al.'s [2] commonly accepted vision that evidence-based practice is the result of the interaction between scientific knowledge, professional experience, and patient values, makes clinical practices the ideal environment for developing and improving methods of treatment, as well as for studying their effectiveness. Several steps can be taken to stimulate research activities within mental healthcare institutes, such as financial facilitation, educating researchers, and organizing collaborations between universities and health care institutes. Barkham et al. [3] stress the importance of, as they call it, 'practice-based evidence' research that determines the relevance of the results from more rigorous, often academy-based, studies in daily practice. As Rohde et al. [4] state "there is general agreement that the best data are those that come from the 'horse's mouth'. In a health-service context, the data which are collected and used by clinicians for the management of their patients are the most reliable." Nevertheless, the number of institutes in the Netherlands that collaborate with universities is limited [5].

A potentially powerful way to stimulate research activities in mental health institutes, including those without a scientific tradition, is to facilitate the process of collecting research data. However, the effort of additional documentation for research or outcome monitoring often conflicts with daily practice, where health professionals have to maintain a high production level and are expected to spend their time with patients, not on documentation. Therefore, it is important to organize the collection of relevant data in such a way that it takes as little time and effort as possible [6]. Moreover, data collection should occur as efficiently as possible, since according to the Helsinki declaration of the WMA, this is the researcher's ethical obligation.

Data analysis for scientific research or quality assessment of care is generally considered an important possible benefit of maintaining electronic patient records [7,8]. Although the importance of the routine measurement of outcomes is often stressed [9], in daily practice it proves to be far from common [10]. One way to generate data is to create large registries of patients and the care provided for specific regions or fields of interest. Examples of such registries in the Netherlands are the Health Information System of the Dutch Mental health care institutes (Zorgis), and databases on youth care and addiction-related treatments. In addition, a number of regional case registries have been constructed, which contain anonymous data on patients and care procedures. A common characteristic of all of these initiatives is that the collected data are more suited for longitudinal epidemiological research on care consumption [11] than for research on the effectiveness and efficacy of treatments, and therefore do not sufficiently support research that aims for evidence-based practice or practice-based evidence. The criticism of Lakeman on the ambitious, nationwide Australian effort to collect routine outcome data [12] is an example of the lack of fit between research database and research aims: "routine standardized outcome measurement in its current form can only provide a crude and narrow lens through which to witness recovery" [13].

Often, the creators of such a multi-purpose database neglect, prior to constructing their database, to consider the questions researchers in mental health organizations actually have, and what data are needed to answer these research questions.

In the patchwork-like structured world of practice research in mental health organizations [14], it is not uncommon that every researcher defines his or her own dataset from scratch. The question then is if a commonality between these individual datasets can be found, which could form the basis for a more generally used minimal dataset. To answer this question, insight into the variety of the data collected for research is needed. Can research data be reduced into a useable minimal data set, or should a variety of research data be accepted and dealt with as a fact of life? In the latter case, a focus on developing a dynamic, highly flexible data collection facility is needed. This article will attempt to answer this question by describing the results of an inventory on what data are collected for research purposes in mental health care organizations.

## Methods

Publications are a suitable source for obtaining insight into the data needed for research. Scientific articles concerning mental health care, however, do not always provide a complete and detailed description of which data have been collected, and which methods were used to do so. In many cases, more data are collected than turn out to be relevant in the context of an article. Besides, not all studies result in traceable publications that meet scientific standards of reporting. This is particularly the case for studies conducted by peripheral institutes, which, in this inventory, are considered an area of special interest. Descriptions of studies in research databases or in annual reports of institutes also provide insufficient detail information. Therefore, in order to get a picture of the data that are used in mental health care facilities, we questioned researchers of completed, pending, or planned studies.

### Tracing of researchers

The following attempts were made to identify researchers in mental health institutes:

• A literature search using Pubmed. Using general psychiatric MESH terms such as 'Mental Health Service' combined with 'Netherlands' almost exclusively lead to studies conducted by universities, not by mental health institutes. A search with the term 'GGZ', the abbreviation generally used for mental health institutes in the Netherlands, produced 54 articles. In 48 of these, the term was found in the Medline section AD, the address information of the author.

• A review of Mental Health Knowledge Centers [15,16], institutes that have a more or less official status in the Netherlands as a center of expertise regarding a specific mental illness, showed that the amount of research performed or coordinated by these centers was extremely varied. Again, most research was performed by large research institutes related to universities.

• A review of online Research databases, such as the Dutch Research Database (NOD) [17], overviews by the Dutch Central Committee on Research involving Human Subjects (CCMO) [18], and Medical research programs subsidized by the Dutch government [19] also showed the same focus on university based studies.

• An inventory of websites of mental health care institutes in the Netherlands [20]. A total of 110 websites were screened for the terms 'scientific' and 'research'. References to these terms were found in 27 of the 110 websites. However, some institutes that are known for their research activities did not present these in any form on their websites.

The attempts did not result in a satisfying overview of research activities, especially those carried out by peripheral institutes. Therefore, we decided to directly approach all the members of the Dutch national mental health organization GGZ-Nederland for the names and e-mail addresses of employees who perform research activities that involved data collection in the period 2000–2005.

### Inventory of data used by researchers

The researchers whose e-mail address was obtained, were asked to list which individual variables (data items not collected using published, validated measurement instruments) were collected about clients and which measurement instruments were used. The data were collected by means of a web form.

The individual variables that were reported were divided in four categories: demographic, diagnostic, intervention, and effect variables. Demographic variables refer to the general properties of the members of the research population other than the nature of illness or complaints. Diagnostic variables are those items that describe the nature and the severity of the illness or complaint, while intervention variables describe the actions that are expected to have a therapeutic result. Finally, effect variables are the variables that express the results of the intervention(s) in the members of the research population.

Regarding the measurement instruments, we asked for the number of times the instrument was used per person. Multiple admissions suggest measurement of a change over time, and therefore the effect of an intervention or program.

Based on the descriptions, we distinguished between effect studies and other studies in order to assess if the institutes' specific demand to evaluate the effects of treatments involved specific variables and instruments.

Three institutes complained that the quantity of their research activities made completing the forms for all studies a time-consuming effort. Some researchers solved this problem by entering the data about more than one study in one form. Because this did not interfere with the purpose of the data collection, these forms were accepted and treated as one study. Other institutes sent an annual report describing the research programs instead of detailed information per study. These institutes were visited to collect additional information in interviews.

## Results

### Researchers and studies

Of the 110 mental health institutes, 87 (79%) responded to the request to send names and e-mail addresses of employees performing scientific research within the institute. Two institutes replied they had active researchers, but chose not to cooperate because it would take too much time to answer our questions; 65 institutes (75% of those responding) provided the names of researchers, and 20 institutes responded that they did not conduct any scientific research activities (23% of the responding institutes).

The 227 employees who were identified as researchers by the institutes were asked to complete the form; 109 (48%) replied. In total, 82 researchers described a total of 92 studies, while 27 (25% of the reactions) responded that they did not perform research in the selected time period or were co-researcher in a study already described by a colleague.

The studies described varied in aim, scope and size. Both large longitudinal studies performed in multiple institutes and small initiatives were included. A large proportion of the studies was reported by smaller institutes; this kind of research cannot be found through other search strategies and is therefore not usually included in the reviews of research activities. Relatively little response came from large research institutes, which were often joint ventures between a mental health care institute and a university.

Based on the descriptions, half of the studies (46) were considered as designed for evaluating the effect of treatment. The other half consisted of epidemiological studies, case descriptions, and validation of measurement instruments.

### Data used by researchers

The number of variables and instruments per domain used in studies are presented in Table 1. The descriptions provided by the researchers reported a total of 124 different variables used in 92 studies. They were reported a total of 357 times, which means every variable is used on average in three studies. The largest number of variables belonged to the diagnostic category, and most agreement existed about demographic variables. More variables were reported from effect studies than from other types of studies. The division of variables across domains was similar for both types of studies. Only one study described effects in terms of variables (not using instruments). The variables that were reported in more than 10% of the studies were date of birth, diagnosis (DSM IV), the type of treatment, gender, ethnic background, and whether or not one participated in a program.

**Table 1:** Number of variables and instruments per domain used in studies

	Domains	Number of different variables/instruments		Use per type of study				Total (average use per variable/instrument)	
						
				Effect (N = 46)		Other (N = 46)			
Variables									
	Demographic	30	24%	74	37%	67	43%	141 (4.8)	39%
	Diagnostic	51	41%	59	30%	47	30%	106 (2.2)	30%
	Treatment/intervention	39	31%	63	32%	43	27%	106 (2.8)	30%
	Effect	4	3%	4	2%	0	0%	4 (1)	1%
	Total	124	100%	200	100%	157	100%	357 (3.1)	100%
Instruments									
	Demographic	0	0	0	0				
	Diagnostic	112	50%	59	25%	78	60%	137 (1.2)	37%
	Treatment/intervention	1	0%	0	0%	1	1%	1 (1)	0%
	Effect	110	49%	177	75%	52	40%	229 (2.1)	62%
	Total	223	100%	236	100%	131	100%	367 (1.6)	100%

A total of 223 different measurement instruments were used in the studies, reported 367 times (Table 1). 112 instruments were characterized as diagnostic, and they were used 137 times, which is an average use of about 1.2 studies per instrument. The 110 instruments that measure effects were used 229 times, which equals an average of two studies per instrument. One single instrument was used to describe the treatment. In effect studies, more instruments were used than in other studies. In 13 studies, three of which were effect studies, no usage of instruments was reported. Effect instruments were primarily applied in effect studies, whereas diagnostic instruments dominated other types of studies. The variety of instruments used was largest in non-effect studies, where 131 reports of instrument use involved 99 different instruments (1.3 studies per instrument). In effect studies, 236 reports of 149 different instruments were noted (1.6 study per instrument).

The averages in Table 1 are influenced by a small number of variables and instruments that are frequently used. Most of the data types are used in only one study: 66% of the variables and 73% of the instruments. Only three variables and two instruments were used in more than ten of the 92 studies. (Table 2)

**Table 2:** Use of variables and instruments

	Number of studies used in	Number of different variables/instruments		Use per type of study				Total use	
						
				Effect (N = 46)		Other (N = 46)			
Variables									
	1	76	61%	39	51%	37	49%	76	21%
	2	15	12%	16	53%	14	47%	30	8%
	3 to 5	21	17%	44	56%	35	44%	79	22%
	6 to 10	9	7%	43	65%	23	35%	66	18%
	>10	3	2%	58	55%	48	45%	106	30%
	Total	124	100%	200	56%	157	44%	357	100%
Instruments									
	1	162	73%	99	61%	63	39%	162	44%
	2	34	15%	41	60%	27	40%	68	19%
	3 to 5	23	10%	62	67%	31	33%	93	25%
	6 to 10	2	1%	13	81%	3	19%	16	4%
	>10	2	1%	20	71%	8	29%	28	8%
	Total	223	100%	235	64%	132	36%	367	100%

The instruments most commonly used were general inventories of symptoms (SCL-90, BDI, CBCL) or patient satisfaction. In order to provide detailed insight into what data are most commonly used in research, the instruments that were reported in more than two studies are presented in Table 3.

**Table 3:** Instruments used in >2 studies

Instrument	Total number of studies
SCL-90 (Symptom Checklist)	16
Tevredenheids thermometer [Client satisfaction thermometer]	12
BDI (Beck Depression Inventory)	8
CBCL (Child Behavior Checklist)^a^	8
CGI (Clinical Global Impression)	5
F-schaal (Functioning level)	5
GAF (Global Assessment of Functioning Scale)	5
HoNOS (Health of the Nation Outcome Scales)	5
PANSS (Positive and Negative Symptom Scale)	5
UCL (Utrecht Coping List)	5
YSR (Youth Self Report)^a^	5
CAN (Camberwell Assessment of Need)	4
CIDI (Composite International Diagnostic Interview)	4
SCID (Structured Clinical Interview for DSM IV diagnosis)	4
SDQ (Strength and Difficulty Questionnaire)	4
TRF (Teachers Report Form)^a^	4
WHOQol Bref (World Health Organization Quality of Life)	4
Alcos-12 (Algemene Competentieschaal) [General Competence Scale]	3
EQ-5D (Euro Qol)	3
HCR-20 (Historical Clinical Risk assessment)	3
LqoLP (Lancashire Quality of Life Profile)	3
OQ-45 (Outcome Questionnaire)	3
PUL (Positieve Uitkomsten Lijst) [Positive Outcome List]	3
Remoralisatieschaal [Remoralization scale]	3
SIG (Schaal voor Interpersoonlijk Gedrag) [Scale for Interpersonal Behavior]	3
WAIS Short Form (Wechsler Adults Intelligence Scale)	3
WAV (Werk Alliantie Vragenlijst) [Work Alliance Questionnaire]	3
WHOQol (World Health Organization Quality of Life)	3

^a ^CBCL, YSR and TRF are closely related instruments, and can be interpreted as one instrument. However, because they were reported separately and are completed by different people, they have been included as separate instruments.

After the received web forms had been processed, several interviews were conducted with research coordinators from institutes that sent annual reports instead of detailed data on specific studies. Data on 20 (22%) of the described studies were obtained using this method. These 20 studies contained 95 (37%) occurrences of the use of an individual variable, and 81 (22%) of an instrument. Four (3% of all reported) of the variables were not reported in the web forms, and 32 (14%) of the instruments were new.

## Discussion

The results show there is a great amount of diversity in the variables and instruments used in mental health research. An important question regarding the data collected is to what extent they give an accurate picture of the reality.

At two points in the design of this inventory, there was a possibility for non-response: (1) after the letter to the institutes has been sent out, and (2) after the e-mail to the researchers. The first question, which inquired after the names of the researchers, was answered by most of the institutes. However, only half of the researchers replied. Besides a general reluctance to answer internet questionnaires [21], a number of explanations were given and deduced from remarks.

Several researchers who did not work with data regarding patients, considered the questions unrelated to their study. Other researchers declined, indicating they had been approached to fill out questionnaires regarding their research too frequently, and were tired of completing surveys about their studies. Whenever a researcher was involved in a large number of studies, completing a questionnaire per study was often considered too much effort. This could explain the lower response rate in larger research centers. Finally, some researchers did not consider the background of the inquiry relevant to their situation, and thus did not feel the need to complete the questionnaire. This finding is contrary to the fact that numerous others expressed the urgent need for improved possibilities for data collection in the care process.

The study was not comprehensive nor entirely representative and the over-representation of studies from peripheral compared to large university institutes may have led to some bias in the results. Despite this, the findings did demonstrate substantial variety in research variables and instruments and unlike many other overviews of research activities, the current study did not overlook smaller projects. The reactions show there is a great deal of scientific activity in smaller organizations (67 of the 87 responding institutes claimed to conduct scientific research). To promote a more evidence-based approach to treatment, it is important that research activities are supported and facilitated where possible.

There is some difficulty in comparing findings across institutes due to the way the data were aggregated. However, it appeared from the data that some institutes may have a preference for particular instruments, an observation that warrants further investigation in the future.

The interviews with research coordinators of larger research institutes that sent an annual report instead of filling out the questionnaire, corrected the possible imbalance to some extent. The data that were used in these institutes added only 3% new variables, which suggests a saturation effect. However, the 14% new instruments that were added indicate that the list of instruments used in research is far from conclusive. The number of instruments used only in a single study could suggest these are more or less idiosyncratic questionnaires. This, however, is not the case; most of the reported instruments could be found in overviews of instruments available in the Dutch language.

The distinction between instruments used to determine a diagnosis and instruments utilized to measure the effects of treatment is based on the assumption that a diagnosis is generally determined once and measuring an effect requires multiple measurements. This differentiation is not based on the content characteristics of all specific instruments, and should only be seen as an attempt to point out to some extent where the variety of the data can be found.

The fact that researchers state that they use an item in their study does not necessarily imply that it is relevant. To determine to what extent the data collected for a study are actually necessary would require asking the researchers which data is used in, and is in fact critical for, the analysis. Within the context of this article, this question has not been answered. We also did not elaborate on the question if or to what extent the number of instruments used in the studies is justified by the unique characteristics of each of them, or if alternatives could have been used as well. Although we have the impression the studies could have been designed using less then 223 instruments, we have not investigated this. Our aim was merely to demonstrate the variety, without making a judgment on the individual choices made in study designs.

The data presented here only uncover a part of the variety in the data collected by the studies. Additional variance results from differences in definitions, coding rules and measurement scales. Age, for instance, can be expressed in years, but also in days or through more generic categories such as child, adult, and elderly. Moreover, the moment in time when a value is documented can provide additional complexity. Values can have multiple occurrences per individual, determined by multiple measurement points in a treatment. This means the actual variance of data used in studies is even larger than suggested by the figures in this article.

Confronted with the variety in data types (both variables and instruments) used in research in mental health organizations, the question arises if this lack of uniformity results from the fact that researchers often define and collect their own data sets, without first considering the availability of other sources, or that their research questions really require the more or less unique sets of data that appear to be the standard. The adoption of routinely used outcome measures has the advantage that results are comparable to other studies. Additionally, using the experience gained through the application of an instrument in multiple studies will also lead to a higher efficiency. Conversely, a disadvantage of standardization could be a scientific stagnation by discouraging the use of promising new instruments [22].

The tendency to collect data specifically for research could be compared to the findings of Snoeker et al. [23] that professionals in mental health care tend to not read the patient record when they meet a patient for the first time. The reasons given for this phenomenon by professionals, such as keeping an open mind to the problem, lack of confidence in existing data, and difficulties in accessing large and poorly organized files, could also apply to researchers.

Electronic records, in the view of Snoeker and many others, have substantial advantages compared to paper files, because data are more easily accessible and can be checked more effectively in terms of quality, partly due to validity checks in the data entry process. This could also be applicable to data collection for research activities.

## Conclusion

There is little commonality among the data used in research in mental health institutes. Only a small set of demographic, diagnostic, and intervention data are, to some extent, shared among the different studies.

The diversity among the different studies shows that restricting research data to a minimum set of variables and instruments will not cover research needs. Therefore, a facility which supports data collection will need to be extremely flexible in order to include both specific data as well as a set of routine data. Furthermore, it needs to be easily maintainable and accessible for researchers, and not require long and cumbersome procedures.

However, more agreement on what variables and instruments to use in relation to research questions is essential in increasing the comparability and therefore the value of scientific results.

## Competing interests

The author(s) declare that they have no competing interests.

## Authors' contributions

JL carried out the data collection, data analysis and drafted the manuscript. JB and PVR contributed to the design of the study and provided comment on the manuscript. All authors read and approved the final version of the manuscript.

## Pre-publication history

The pre-publication history for this paper can be accessed here:


